# Dystrophia Adiposa Corneae: An Out-of-the-Box Diagnosis

**DOI:** 10.7759/cureus.67580

**Published:** 2024-08-23

**Authors:** Tushar Agrawal, Pranav More, Radhika Paranjpe, Vishakha Vatkar, Pradipta P Potdar

**Affiliations:** 1 Ophthalmology, Dr. D. Y. Patil Medical College, Hospital and Research Centre, Dr. D. Y. Patil Vidyapeeth (Deemed to be University), Pune, IND

**Keywords:** crystalline keratopathy, dyslipidemia, keratopathy, fatty degeneration, lipid deposition

## Abstract

Dystrophia adiposa corneae is a degenerative corneal disorder characterised by fatty deposition, leading to corneal opacity and reduced visual acuity. We report a case of an Indian male in his early 40s with bilateral peripheral deep lipid deposition in the corneal stroma, beginning in an arcuate fashion and progressing to an annular-shaped structure. Furthermore, cholesterol crystals, as well as crystalline structures, were seen without any neovascularisation. We confirmed these findings on anterior segment optical coherence tomography (AS-OCT). We also noticed an increase in central corneal thickness (CCT), and on systemic workup, the patient was diagnosed with mild dyslipidaemia. This case highlights the need for an out-of-the-box diagnosis of a rare keratopathy, which can be vision-threatening and needs to be differentiated from its mimickers.

## Introduction

Dystrophia adiposa corneae, also known as xanthomatosis, lipid keratopathy, and lipidosis corneae, was first described by Cogan and Kuwabara in 1958 as a lipid exudation adjacent to vascularisation occurring due to trauma or inflammation [1,2].

It is a rare ophthalmological condition with an overall prevalence of 0.01% and a higher prevalence in individuals of lower socioeconomic status (0.02%) [3]. It is characterised by lipid deposition leading to corneal opacity due to lipid deposition. The etiological classification includes primary and secondary forms. Primary, or idiopathic, is usually bilateral, associated with no prior inflammation or vascularisation, and with normal lipid levels, while secondary is usually unilateral, associated with trauma, iatrogenic damage, or previous corneal inflammation. Lipid accumulation occurs due to reduced lipid metabolism or increased lipid production. If neo-vascularisation occurs, it can present as superficial stromal, deep stromal, or as vascular pannus [4]. These lesions are progressive and can affect the visual axis, leading to reduced visual acuity. The opacities that are formed can histologically resemble extracellular as well as intracellular cholesterol crystals, and the features can be visualised by Oil Red O and Sudan Black B on frozen tissue. If a frozen section is not available, it can also be visualised on haematoxylin and eosin-stained slides.

Reports have shown that these opacities may be associated with hypercholesterolemia and manifestations of arcus lipoides and xanthelasma [2]. Hence, this condition needs to be differentiated from its mimickers, such as corneal arcus, Schnyder's corneal dystrophy, systemic dyslipoproteinemias - including familial lecithin-cholesterol acyltransferase disease (FLD), fish-eye disease (FED), and Tangier disease (TD) - and others. There can also be a fan-shaped plaque present in active keratitis [5]. Corneal arcus is an ocular lipid deposition characterised by cholesterol as well as phospholipid accumulation in the peripheral cornea. It is a normal ageing process, bilateral, occurring over 50 years without inflammation or cellular damage [6]. Schnyder's corneal dystrophy, on the other hand, is associated with cholesterol and phospholipid deposition and vision loss. It usually occurs in the first few decades of life, with central haze, later progressing peripherally and involving the anterior stroma and Bowman's membrane. FLD, FED, and TD manifest with low plasma high-density lipoprotein (HDL) levels and corneal clouding. FLD is characterised by bilateral corneal opacities comprised of granular dots limited to the anterior stroma. FED is characterised by partial LCAT deficiency with dot-like opacities in all layers except the epithelium. TD is characterised by HDL deficiency, presenting with corneal clouding, neuropathy, cardiovascular disease, and more. Additionally, Terrien marginal degeneration is a non-inflammatory thinning of the peripheral cornea, characterised by corneal furrowing beginning superiorly, followed by superficial vascularisation and lipid deposition at the leading edge of the pannus [7].

## Case presentation

A healthy male in his early 40s came to our Ophthalmology Outpatient Department complaining of greyish-white discolouration in both eyes, with gradual, painless deterioration of vision in both eyes over two years. Moreover, he reported a history of hypertension for the past year, for which he was on treatment. His best-corrected visual acuity (BCVA) was 20/40 with +0.75 D sph and -2.0 D cyl at 10 degrees in the right eye, and 20/60 in the left eye with -0.50 D sph and -1.0 D cyl at 90 degrees, respectively. Intraocular pressures were normal. The patient also had a history of hospitalisation one year ago for diplopia and was diagnosed with left lateral rectus paresis, for which he was started on systemic steroids, i.e., intravenous methylprednisolone 1 g/day for one week. He also had a history of deep keratitis in both eyes six months ago, for which he was treated with topical steroids three times a day, with a tapering dose. He had no history of trauma, infection, or use of drugs like fluoroquinolones. On slit lamp examination, the right eye revealed disk-like annular lesions with whitish shiny deposits at the temporal periphery in the corneal stroma, and in the upper half of the corneal stroma, appearing deeper in the left eye. No corneal blood vessels or any other ocular abnormality were noted.

**Figure 1:**
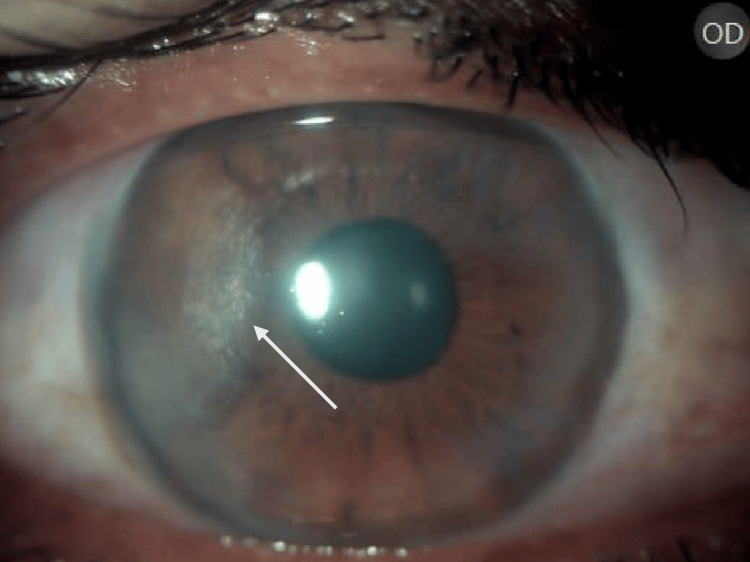
Right eye showing crystalline deposits on the cornea

Whitish, cream-coloured crystalline deposits in a disciform manner, temporally on the cornea (Figure 1).

**Figure 2:**
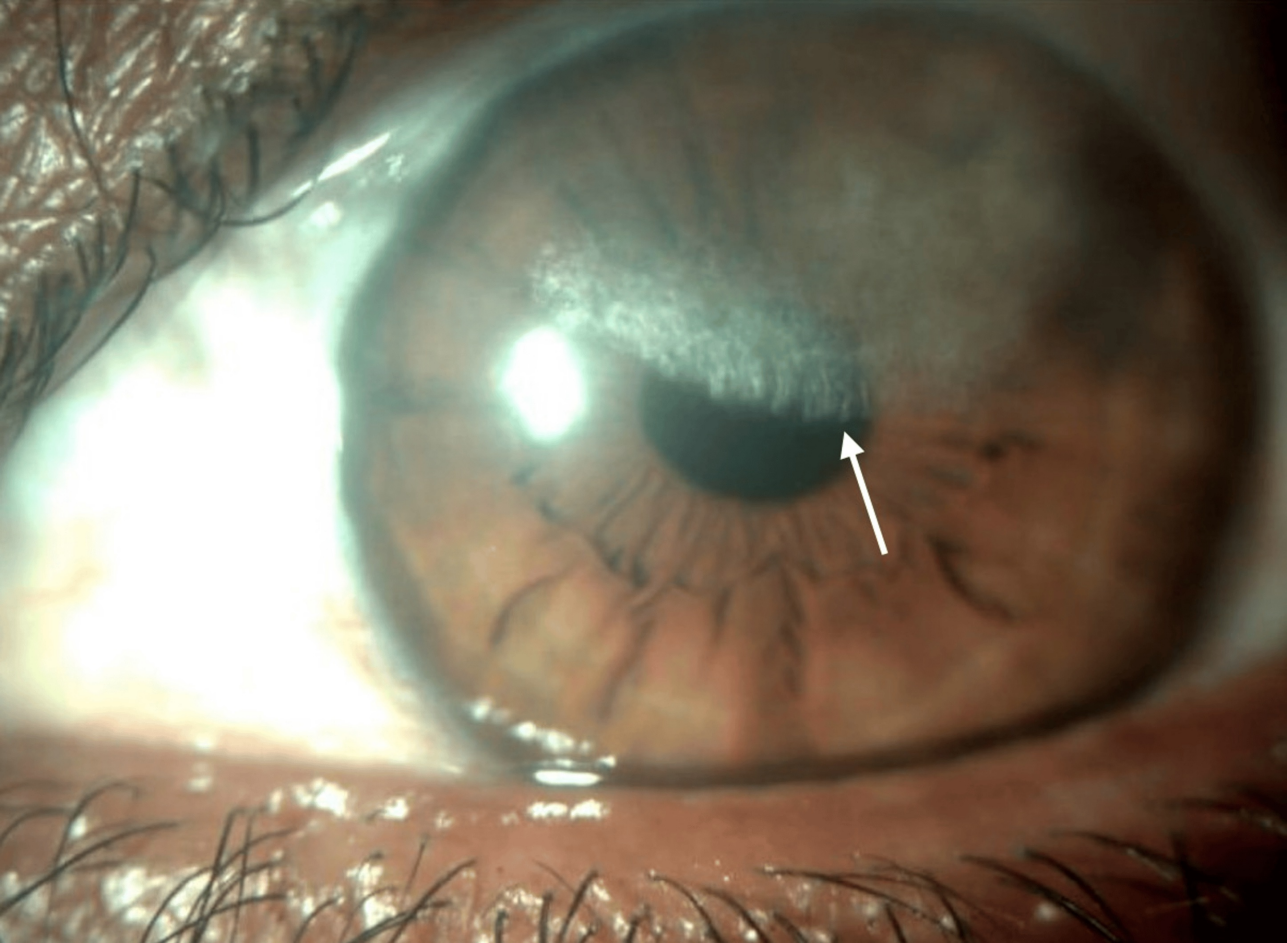
Left eye showing crystalline deposits on the cornea

Whitish, fan-like crystalline opacification in a disciform manner, superiorly on the cornea, progressing towards the visual axis (Figure 2).

The corneal epithelium was intact, with negative fluorescein staining and intact corneal sensations. The rest of the anterior segment examination was within normal limits. Anterior segment optical coherence tomography (AS-OCT) was done.

**Figure 3:**
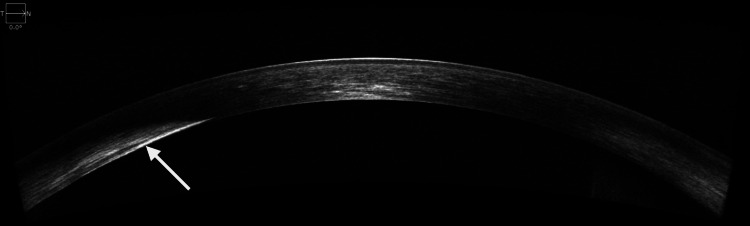
Anterior segment optical coherence tomography (AS-OCT) of the right eye This is an image of the right eye AS-OCT, showing a whitish, thickened area temporally in the posterior stroma, while appearing normal nasally.

AS-OCT of the right eye revealed zones of hyperreflectivity at the level of the stroma, suggestive of posterior stroma involvement temporally (Figure 3).

**Figure 4:**
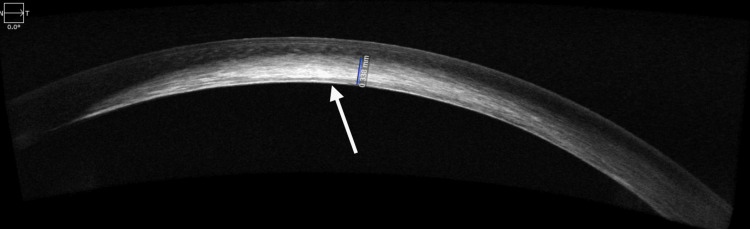
Anterior segment optical coherence tomography (AS-OCT) of the left eye This is an image of the left eye AS-OCT, showing a whitish, thickened area centrally in the posterior stroma, as well as in the mid-stroma, while appearing normal nasally and temporally.

AS-OCT of the left eye revealed zones of hyperreflectivity at the level of the stroma, suggestive of the involvement of almost the entire stroma, including the visual axis (Figure 4).

Corneal thickness at the site of the lesion in the right eye was 690 micrometres and could not be recorded in the left eye. Fundus examination was within normal limits in both eyes.

The patient was counselled for the general examination, which revealed a blood pressure of 160/90 mmHg. The lipid profile revealed a total cholesterol level of 225 mg/dL (normal < 200 mg/dL), triglyceride levels of 238 mg/dL (normal < 152 mg/dL), HDL levels of 24 mg/dL (normal > 40 mg/dL in men), very low-density lipoprotein (VLDL) of 30 mg/dL (normal < 30 mg/dL), and low-density lipoprotein (LDL) cholesterol levels of 171 mg/dL (normal < 100 mg/dL). Serum homocysteine levels were 25.67 mmol/L (normal for males are 5.08 to 15.39 mmol/L). We concluded that hypertriglyceridemia was present in his lipid profile and offered him a cardiology examination to control his lipids. He underwent two-dimensional echocardiography, which revealed left ventricular hypertrophy and was started on cholesterol-lowering drugs, which could halt the progression.

A diagnosis of dystrophia adiposa corneae in both eyes was made. Informed consent from the patient was obtained to report this case. The patient was started on lubricating eye drops and was informed that he might require a corneal transplant in a few years, if the opacity increases to involve the central part of the visual axis, to improve his BCVA.

## Discussion

Dystrophia adiposa corneae, also known as lipid keratopathy, is an out-of-the-box diagnosis, as it typically occurs in elderly individuals but, in our case, was seen in a young individual. It is a rare disorder characterised by lipid deposits in the cornea, occurring primarily without vascularisation, as in our case, or secondarily with the presence of corneal blood vessels. This condition needs to be diagnosed early and intervened upon; otherwise, it could lead to a vision-threatening condition. The pathogenesis still remains unclear, but, according to us, corneo-scleral microvascular changes with incompetent limbal blood vessels may account for corneal lipid infiltration [8].

Ghanem et al. reported two patients with bilateral peripheral deep stromal lipid deposits progressing to a complete annular shape, with neovascularisation where cholesterol crystals were seen in confocal microscopy. They observed insidious progression of infiltrates for more than a decade, and penetrating keratoplasty was not required [4].

There have been various studies done on the secondary variant of dystrophia adiposa corneae, where subconjunctival bevacizumab injection once monthly leads to a reduction in neovascularisation, as documented by Oh et al. [9], and by Hussain and Savant, where one subconjunctival bevacizumab injection led to the cessation of progression or some degree of regression of lipid deposition [10].

Loeffler and Seifert reported a case of bilateral disease with bulging towards the anterior chamber, having intracellular and extracellular lipid deposition, and mild hypertriglyceridemia, which had a systemic association, i.e., a tumour in the pituitary gland [11]. Croxatto et al. reported a case of bilateral disease with idiopathic cholesterol deposits but normal cholesterol levels, which had a systemic association, namely a venereal disease for which the patient was treated [12]. Similarly, in our case, there was bilateral involvement with hypertriglyceridemia, and the patient also had left ventricular hypertrophy, for which he was started on cholesterol-lowering drugs.

## Conclusions

Dystrophia adiposa corneae is a disease characterised by cholesterol deposits, sometimes accompanied by corneal neovascularisation, leading to opacification of the cornea and reduced visual acuity. The cause in idiopathic cases still remains unknown but is bilateral, while the other form is related to trauma or a disease process and is unilateral. There have been many treatment options tried for this condition, such as steroids, anti-vascular endothelial growth factor (VEGF) agents, photodynamic therapy, laser treatment, and, if nothing works, keratoplasty. However, further studies must be performed to identify reliable, affordable, and accessible options for patients.
